# MicroRNA-200c inhibits the metastasis of non-small cell lung cancer cells by targeting ZEB2, an epithelial-mesenchymal transition regulator

**DOI:** 10.3892/mmr.2021.12212

**Published:** 2021-06-08

**Authors:** Aihong Jiao, Minghua Sui, Liangming Zhang, Ping Sun, Dongmei Geng, Weiwei Zhang, Xiuwen Wang, Junxia Li

Mol Med Rep 13: 3349-3355, 2016; DOI: 10.3892/mmr.2016.4901

Following the publication of this paper, it was drawn to the Editors' attention by a concerned reader that certain of the cell Transwell assay data in the article (featured in Figs. 2C and 4C) were strikingly similar to data appearing in different form in other articles by different authors at different research institutions, which were already under consideration for publication or had already been published elsewhere at the time of the present article's submission [C. Lai *et al*, ‘MicroRNA-133a inhibits proliferation and invasion, and induces apoptosis in gastric carcinoma cells via targeting fascin actin-bundling protein 1’, Mol Med Rep 12: 1473-1478, 2015; and Y. Shi *et al*, ‘MicroRNA-204 inhibits proliferation, migration, invasion and epithelial-mesenchymal transition in osteosarcoma cells via targeting Sirtuin 1’, Oncol Rep 34: 399-406, 2015].

Owing to the fact that the contentious data in the above article had already appeared in different form in other articles prior to its submission to *Molecular Medicine Reports*, the Editor has decided that this paper should be retracted from the Journal. The authors did not reply to indicate whether or not they agreed with the retraction of the paper. The Editor apologizes to the readership for any inconvenience caused.

